# Elevation of Proinflammatory Cytokine HMGB1 in the Synovial Fluid of Patients With Legg‐Calvé‐Perthes Disease and Correlation With IL‐6

**DOI:** 10.1002/jbm4.10429

**Published:** 2020-12-03

**Authors:** Nobuhiro Kamiya, Harry KW Kim

**Affiliations:** ^1^ Center for Excellence in Hip Scottish Rite for Children Dallas TX USA; ^2^ Department of Orthopedic Surgery University of Texas Southwestern Medical Center Dallas TX USA; ^3^ Faculty of Budo and Sport Studies Tenri University Nara Japan

**Keywords:** HIGH‐MOBILITY GROUP BOX 1, INTERLEUKIN‐6, ISCHEMIC OSTEONECROSIS, LEGG‐CALVÉ‐PERTHES DISEASE, PROINFLAMMATORY CYTOKINE

## Abstract

Legg‐Calvé‐Perthes disease (LCPD) is a childhood ischemic osteonecrosis (ON) of the femoral head associated with the elevation of proinflammatory cytokine interleukin‐6 (IL‐6) in the synovial fluid. Currently, there is no effective medical therapy for patients with LCPD. In animal models of ischemic ON, articular chondrocytes produce IL‐6 in response to ischemic ON induction and IL‐6 receptor blockade improves bone healing. High‐mobility group box 1 (HMGB1) is a damage‐associated molecular pattern released from dying cells. In addition, extracellular HMGB1 protein is a well‐known proinflammatory cytokine elevated in the synovial fluid of patients with rheumatoid arthritis and osteoarthritis. The purpose of this study was to investigate IL‐6–related proinflammatory cytokines, including HMGB1, in the synovial fluid of patients with LCPD. Our working hypothesis was that HMGB1, produced by articular chondrocytes following ischemic ON, plays an important role in IL‐6 upregulation. Here, HMGB1 protein levels were significantly higher in the synovial fluid of patients with LCPD by threefold compared with controls (*p* < 0.05), and were highly correlated with IL‐6 levels (Pearson correlation coefficient 0.94, *p* < 0.001, *R* ^2^ = 0.87). In the mouse model of ischemic ON, both HMGB1 gene expression and protein levels were elevated in the articular cartilage. In vitro studies revealed a significant elevation of HMGB1 and IL‐6 proteins in the supernatants of human chondrocytes exposed to hypoxic and oxidative stresses. Overexpressed HMGB1 protein in the supernatants of chondrocytes synergistically increased IL‐6 protein. Silencing *HMGB1* RNA in human chondrocytes significantly repressed inteleukin‐1β (IL‐1β) gene expression, but not IL‐6. Further, both IL‐1β and tumor necrosis factor‐α (TNF‐α) protein levels in the synovial fluid of patients with LCPD were significantly correlated with IL‐6 protein levels. Taken together, these results suggest that proinflammatory cytokines, HMGB1, tumor necrosis factor‐α (TNF‐α), and IL‐1β, are significantly involved with IL‐6 in the pathogenesis of LCPD. This study is clinically relevant because the availability of multiple therapeutic targets may improve the development of therapeutic strategy for LCPD. © 2020 The Authors. *JBMR Plus* published by Wiley Periodicals LLC on behalf of American Society for Bone and Mineral Research.

## Introduction

Legg‐Calvé‐Perthes disease (LCPD) is a childhood form of ischemic ON of the femoral head, which afflicts 1 in 740 boys and 1 in 3500 girls between the ages of 2 and 14 years.^(^ ^1^, ^2^ ^)^ Currently, there is no effective medical therapy to treat patients with LCPD. The hip disorder can produce a permanent deformity of the femoral head and early osteoarthritis.^(^ ^3^, ^4^, ^5^ ^)^ Although the disruption of the blood supply to the femoral head produces femoral head necrosis,^(^ ^6^, ^7^, ^8^, ^9^, ^10^ ^)^ the etiology of the vascular disruption and the pathophysiology of the disease condition are largely unknown.

High‐mobility group box 1 (HMGB1) was initially reported as an intracellular DNA‐binding protein.^(^ ^11^ ^)^ Currently, the HMGB1 protein is known to translocate to extracellular space in immune cells (ie, active secretion) and dying cells (ie, passive release).^(^ ^12^, ^13^ ^)^ The extracellular HMGB1 protein functions as a proinflammatory cytokine^(^ ^14^, ^15^, ^16^ ^)^ and a prototype of damage‐associated molecular pattern^(^ ^17^, ^18^ ^)^ with a positive feedback loop by synergistically activating other proinflammatory cytokines, such as IL‐6, IL‐1β, and TNF‐α.^(^ ^19^, ^20^, ^21^, ^22^, ^23^ ^)^ In rheumatoid arthritis (RA), a chronic inflammatory autoimmune disorder, HMGB1 protein is actively secreted by immune cells including synovial cells and macrophages at the site of joint inflammation and is elevated in the synovial fluid.^(^ ^22^, ^23^, ^24^ ^)^ In osteoarthritis (OA), HMGB1 protein is passively released from the damaged articular cartilage.^(^ ^25^, ^26^ ^)^ Significant associations of HMGB1 protein in the synovial fluid with OA severity have been reported.^(^ ^27^, ^28^ ^)^ In addition, HMGB1 protein injection into a normal joint has been found to induce arthritis in 80% of mice.^(^ ^29^ ^)^

Chronic synovitis of the affected hip joint is a common feature of LCPD.^(^ ^30^, ^31^ ^)^ Very little research, however, has been geared towards delineating the mechanisms underlying the chronic hip synovitis in LCPD. Our MRI studies showed a significant increase in synovitis and synovial effusion in the early stages of LCPD.^(^ ^32^, ^33^ ^)^ Intriguingly, one of the proinflammatory cytokines, IL‐6, was significantly elevated in the synovial fluid of the affected hip (500 to 1000 pg/mL) compared with controls (<30 pg/mL).^(^ ^32^ ^)^

In pathologic conditions, IL‐6 may have a procatabolic effect on bone metabolism by promoting osteoclast activity (ie, bone resorption), while suppressing osteoblast activity (ie, bone formation).^(^ ^34^, ^35^, ^36^, ^37^, ^38^ ^)^ Using animal models of ischemic osteonecrosis (ON), we recently reported that IL‐6 is produced by articular chondrocytes,^(^ ^39^, ^40^ ^)^ and that IL‐6 receptor blockade preserves articular cartilage and improves bone healing.^(^ ^39^ ^)^ IL‐6 is known to be induced by HMGB1 in synovial cells,^(^ ^41^ ^)^ and HMGB1 binds to IL‐1β,^(^ ^19^, ^42^ ^)^ suggesting that HMGB1 works upstream of IL‐6 by interacting with IL‐1β.

Proinflammatory cytokines HMGB1 and IL‐6 have been studied in chronic inflammatory diseases including OA and RA; however, little is known about their roles in ischemic ON. In addition, the relationship between IL‐6 and HMGB1 in chondrocytes is largely unknown. The purpose of this study was to investigate IL‐6 related proinflammatory cytokines including HMGB1 in the pathogenesis of LCPD. Our working hypothesis was that HMGB1 is produced by articular chondrocytes following ischemic ON, and that HMGB1 plays an important role in activating IL‐6 through IL‐1β.

## Subjects and Methods

This study was approved by the local institutional review board (IRB) at University of Texas Southwestern Medical Center.

### Synovial fluid collection from patients with LCPD for Western blot analysis and protein concentration

Synovial fluid samples were obtained from patients with LCPD at the time of hip arthrography using an 18G needle and fluoroscopic guidance. The hip joints were aspirated without prior injection of contrast solution or any dilution. Because we were not able to obtain normal synovial fluid of the unaffected hip, we used synovial fluid obtained at the time of hip arthrography in patients with hip dysplasia or impingement as controls. Synovial fluid samples were centrifuged to remove cells, and the supernatant was stored at −20°C for analysis later.

For the Western blot analysis, 5 μL of synovial fluid (control: *n* = 3; LCPD: *n* = 5) was diluted with sample buffer up to 60 μL. Then, 5 μL was loaded per each lane on 10% SDS‐PAGE gels. The obtained blot was subsequently incubated with primary antibodies for high mobility group box chromosomal protein 1 (HMGB1; Ab11354, 1:100; Abcam, Cambridge, MA, USA) and α‐actin (1:500; Cell Signaling Technology, Beverly, MA, USA) and secondary peroxidase anti‐mouse (1:1000; Amersham Biosciences, Piscataway, NJ, USA) and anti‐rabbit (1:5000, Amersham Biosciences) antibodies, respectively. For detection, we used the ECL Plus Western Blotting Detection System (Amersham Biosciences). The optical density levels of Western bands were measured using ImageJ software (NIH, Bethesda, MD, USA; https://imagej.nih.gov/ij/), and the correlation between the density and protein concentration per each sample was analyzed.

Protein concentrations of IL‐6, IL1‐β, and TNF‐α in the synovial fluid of patients with LCPD (*n* = 13) were previously measured at the UT Southwestern Medical Center Core facility (Dallas, TX, USA) as we reported in our earlier work.^(^ ^32^ ^)^ In short, human synovial fluid samples were treated with hyaluronidase (4 mg/mL; Sigma‐Aldrich, St. Louis, MO, USA) at 37°C for 1 hour in a shaker, and diluted in the sample diluent with 0.5% BSA as a final concentration.^(^ ^43^ ^)^ After the incubation, samples were centrifuged at 1000*g* for 5 minutes; the supernatant was used for the multicytokine assay kit in duplicates (catalog no.: M50–0KCAF0Y; Bio‐Rad Laboratories, Hercules, CA, USA). Protein concentration of HMGB1 in the synovial fluid of patients with LCPD (n = 8) and control (n = 5) was measured without the treatment of hyaluronidase following the instruction using the HMGB1 ELISA kit (IBL International, Hamburg, Germany).

### Mice and ischemic induction of distal femur

The animal protocol for this study was approved by the Institutional Animal Care and Use Committee of the University of Texas Southwestern Medical Center. In the ON group (*n* = 8), the ischemic induction of the right distal femur was surgically introduced as reported previously.^(^ ^44^ ^)^ In short, skeletally immature juvenile male mice (ie, 6‐week‐old C57BL/6) were anesthetized with isoflurane. Under a microscope (×6 to ×40), the four blood vessels supplying the right distal femoral epiphysis (ie, a branch of a popliteal and branches of the medial, central, and lateral genicular vessels) were identified and cauterized using microsurgical instruments. In the sham group (*n* = 8), the four vessels of the right distal femur were identified, but not cauterized. The right side received either sham or ON surgery (ie, sham or ON); the left side received no surgery (ie, contralateral unoperated side). No adverse events were observed by the surgical technique throughout this study. We used 20 mice in total.

### Protein purification from mouse cartilage and chondrocytes for Western blot analysis

Seven days after the surgery, articular cartilage was collected and lysed with a lysis buffer (0.1M potassium phosphate, 1mM DTT, and 0.2% Triton X‐100) on ice for 30 minutes, then centrifuged down to collect the supernatants. Protein concentrations in the supernatants were determined using a bicinchoninic acid protein assay kit (Pierce Biotechnology, Rockford, IL, USA). Proteins were separated on 10% SDS‐PAGE gels and transferred to a polyvinylidene fluoride membrane followed by a Western blot analysis. In brief, 5% milk in TBS containing 0.1% Tween 20 was used to block the nonspecific binding. The blot was subsequently incubated with primary antibodies for HMGB1 and β‐actin followed by secondary antibodies.

After 48 hours of the transfection, mock‐transfected human chondrocytes, as well as HMGB1‐transfected human chondrocytes, were collected and processed for protein extraction as mentioned above. Western blot was incubated with primary antibodies against β‐actin (1:2000) and Flag (1:1000; Santa Cruz Biotechnology, Santa Cruz, CA, USA) to detect HMGB1, followed by the secondary peroxidase anti‐rabbit antibody.

### Histological analysis

Immediately after the sacrifice at the endpoint, mouse legs were harvested and fixed with 10% formalin for 5 days. The specimens were decalcified with 10% EDTA for 5 days. Bones were embedded in paraffin and cut at 4‐μm thickness. For immunostaining, a mouse monoclonal antibody against HMGB1 (1:100, ab11354; Abcam) was used, followed by the secondary antibody. The antibody binding was visualized with 3, 3‐diaminobenzidine tetrahydrochloride before counterstaining with hematoxylin.

### Primary human chondrocytes and cell lines

We previously isolated primary chondrocytes from human articular cartilage, obtained from a surgical discard at the time of surgery (IRB Protocol Study ID: STU 012011–114).^(^ ^45^ ^)^ In short, the cartilage was digested with collagenase type I (1mg/mL; Sigma‐Aldrich) and Dispase II (2mg/mL; Roche Applied Science, Indianapolis, IN, USA) in an alpha‐minimum essential medium (α‐MEM; Invitrogen, Carlsbad, CA, USA) overnight. Cells were centrifuged and resuspended with fresh α‐MEM containing 20% FBS and antibiotics (100IU/mL penicillin and 100μg/mL streptomycin) at 37°C, 5% CO_2_. Cells were expanded in α‐MEM containing 10% FBS and defined as P1 (passage 1). P2 to P4 cells were used for all experiments under normoxia (21% oxygen) and hypoxia (1% oxygen). Human embryonic kidney 293 and mouse chondrogenic ATDC5 cells were obtained from American Type Culture Collection (ATCC, Manassas, VA, USA) and cultured in DMEM (Sigma‐Aldrich) containing 10% FBS and Ham's F12 (1:1; Sigma‐Aldrich) containing 5% FBS, respectively.

### Hydrogen peroxide addition to chondrocyte culture and ELISA for IL‐6 and HMGB1

Primary human chondrocytes were seeded into a 12‐well plate at 2 × 10^5^ cells per well and incubated for 24 hours. Hydrogen peroxide (H_2_O_2_; 0–200μM; Sigma‐Aldrich) was added to the culture for 24 hours, and supernatants were collected. ELISA kits for human IL‐6 (Invitrogen, Camarillo, CA, USA) and HMGB1 (IBL International, Hamburg, Germany) were used to determine IL‐6 and HMGB1 protein levels in the culture supernatants from primary human chondrocytes treated with various conditions (ie, H_2_O_2_, short interfering RNA [siRNA], and HMGB1 plasmid).

### RNA isolation and real‐time RT‐PCR

The total RNA was isolated from the mouse articular cartilage of distal femur at postsurgery day 1 and cells from various conditions (ie, 293 cells, ATDC5 cells, primary human chondrocytes) using a TRIsol reagent (Invitrogen) and RNA isolation kit (QIAGEN, Germantown, MD, USA), respectively. cDNA was synthesized using the SuperScript Preamplification System (Invitrogen). PCR reactions, data quantification, and analysis were performed according to the manufacturer's standard protocol for the TaqMan gene expression assays using the ABI PRISM 7500 sequence detection system (Applied Biosystems, Rotkreuz, Switzerland). The reaction volume was 20 μL per well in 96‐well plates. Predesigned primers for mouse or human HMGB1, IL‐6, and IL‐1β were used. The target quantity was normalized to an endogenous HSP90 expression. All measurements were performed in triplicate and analyzed using the 2^‐ΔΔ*C(t)*^ method.^(^ ^46^ ^)^

### HMGB1 overexpression in chondrocytes

The expression vector pcDNA3.1 Flag hHMGB1 plasmid was kindly gifted from Dr. Noboru Taniguchi.^(^ ^47^ ^)^ For Western blot analysis and RT‐PCR, 4 × 10^5^ cells and 2 × 10^5^ cells were seeded on 6‐well plates and 12‐well plates, respectively. Cells were transfected with the HMGB1 plasmid or pcDNA3.1 alone (mock) at an amount of 1.4 to 2.2μg of DNA in a serum‐free condition. For transfection reagent, Fugene 6 (Promega, Madison, WI, USA) and Lipofectamine 2000 (Invitrogen) were used with Opti‐MEM (Invitrogen) for 293 cells and chondrocytes (ie, ATDC5, human primary chondrocytes), respectively. After 48 hours of transfection, the cells and culture supernatants were harvested for analysis.

### siRNA interference for HMGB1 using human chondrocytes

Predesigned siRNA oligonucleotide targeting human *HMGB1* (siGENOME) and a control scrambled RNA oligonucleotide targeting a sequence not sharing homology with the human genome (Accell nontargeting siRNA) were purchased (Thermo Scientific Dharmacon, Lafayette, CO, USA). The human primary chondrocytes were grown in a normal medium (α‐MEM containing 10% FBS) for 24 hours to 50% to 60% confluence and were transfected with siRNAs (final 100nM and 10nM) and control scrambled RNA (final 100nM) using the Lipofectamine RNAiMAX transfection reagent (Invitrogen). The culture medium was replaced with OPTI‐MEM (Invitrogen) and suspension was added dropwise onto the cells. After 5 hours of transfection, the culture medium was recovered to the normal medium. After 48 hours of transfection, cells were harvested for each assay. We optimized the condition so that the *HMGB1* expression was about 85% knocked down as determined by qRT‐PCR.

### Statistical analysis

Data are expressed as mean ± SD. In multiple groups, ANOVA and Tukey's honest significant difference tests were used. Between two groups, a two‐tailed Student's *t* test was used. A *p* value <0.05 was considered statistically significant. For the assessment of correlation of the two groups indicated, Pearson product–moment correlation coefficient was determined and a *p* value <0.01 was considered significant. A contribution ratio was expressed as *R* ^2^.

## Results

### Increased HMGB1 protein in the synovial fluid of patients with LCPD

**Fig 1:**
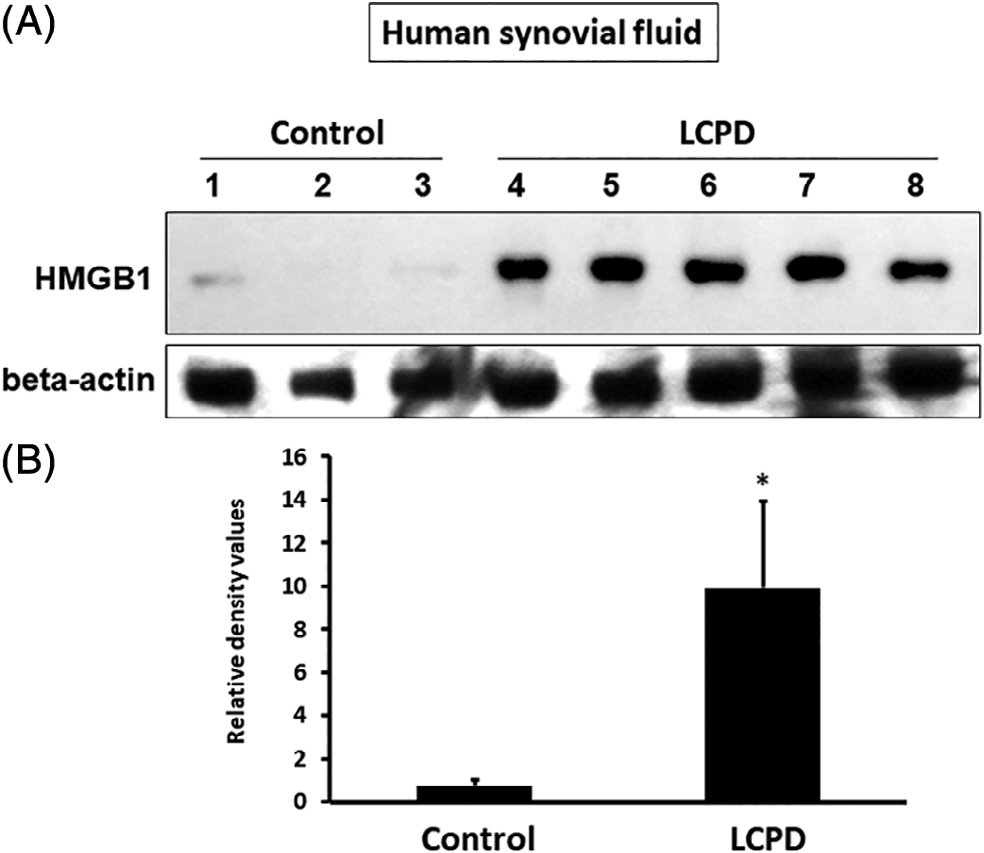
Increase of high‐mobility group box 1 (HMGB1) protein levels in synovial fluid of patients with Legg‐Calvé‐Perthes disease (LCPD). (*A*) HMGB1 protein levels were much higher in the LCPD group compared with the control group (control: #1–3; LCPD: #4–8). (*B*) The integrated density values of each sample from Western blot were measured. The density in the LCPD group was significantly higher than that in the control group. **p < 0.01 (control: *n* = 3; LCPD: *n* = 5; the density of #1 was set as 1.0).

**Fig 2:**
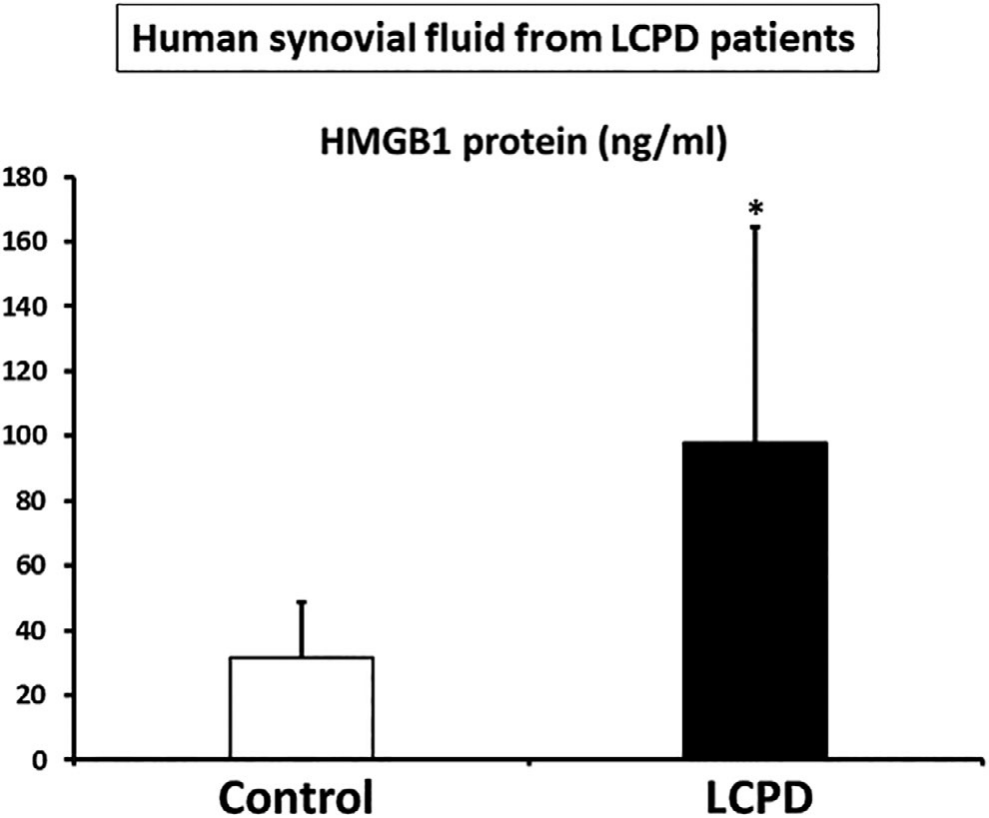
Increased high‐mobility group box 1 (HMGB1) protein in synovial fluid of patients with Legg‐Calvé‐Perthes disease (LCPD). HMGB1 protein levels (ng/mL) in synovial fluid were measured. The mean HMGB1 value was threefold higher in patients with LCPD compared with that in control patients. *p < 0.05 (control: *n* = 5; LCPD: *n* = 8).

We performed Western blot analysis (control: *n* = 3; LCPD: *n* = 5) and found that the resulting optical density of HMGB1 protein was significantly higher in the synovial fluid of patients with LCPD than that of controls (relative HMGB1 values: LCPD 9.91 ± 4.04 versus control 0.77 ± 0.27, *p* < 0.01; Fig. 1*A*,*B* **)**. In addition, the mean HMGB1 value was threefold higher in the patients with LCPD compared with that in the control patients (*p* < 0.05; Fig. 2 **)**.

### Positive correlation of HMGB1 with IL‐6 in the synovial fluid of patients with LCPD

**Fig 3:**
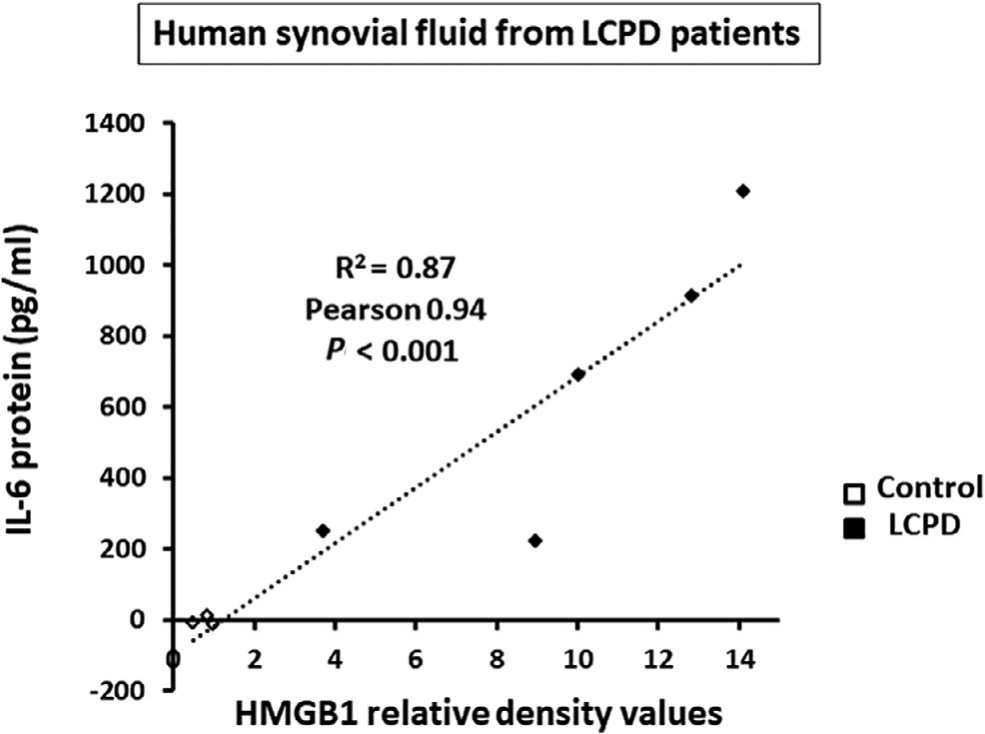
Positive correlation between high‐mobility group box 1 (HMGB1) and IL‐6 protein levels in synovial fluid of patients with Legg‐Calvé‐Perthes disease (LCPD). IL‐6 protein levels (pg/mL) were measured using synovial fluid shown in Fig. 1. The correlation of IL‐6 protein values and the HMGB1 relative density values is shown. Each scatter plot shows one human sample. There is a high and statistically significant correlation between the values of IL‐6 and HMGB1 proteins (Pearson correlation coefficient 0.94, *p* < 0.001, *R* ^2^ = 0.87; *n* = 8).

The protein levels of IL‐6 in the synovial fluid was significantly and highly correlated with the relative density values of HMGB1 protein obtained from Western blot analysis as shown in Fig. 1 (Pearson correlation coefficient 0.94, *p* < 0.001, *R* ^2^ = 0.87; *n* = 8; Fig. 3 **)**. In these samples, the mean concentration of IL‐6 protein (pg/mL) was significantly higher in the synovial fluid of patients with LCPD compared with that of controls (LCPD: 658.7 ± 425.3, *n* = 5 versus control: −3.8 ± 13.2, *n* = 3, *p* = 0.025).

### Increased HMGB1 protein in the articular cartilage using an experimental mouse model of juvenile ischemic ON

**Fig 4:**
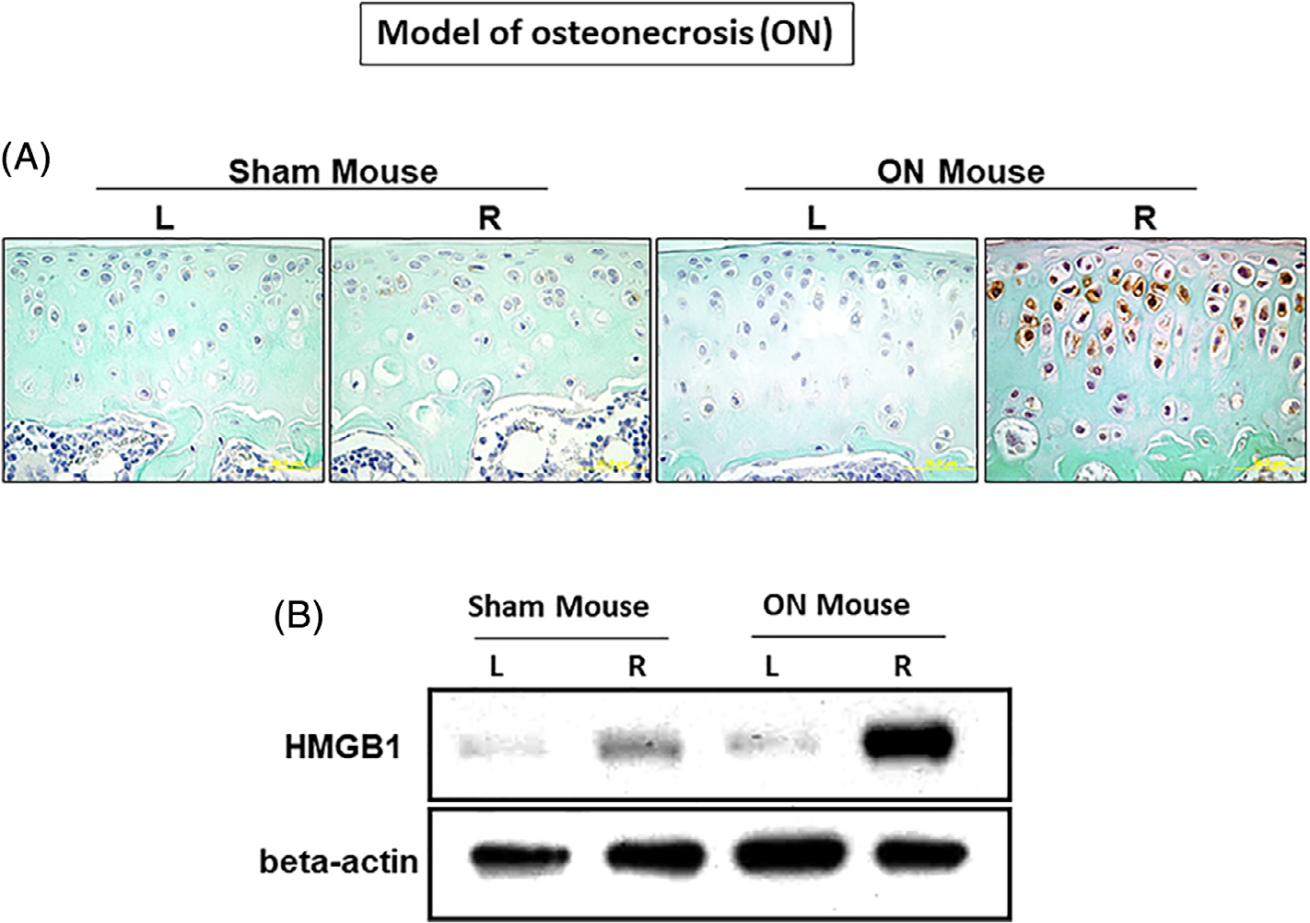
Increased high‐mobility group box 1 (HMGB1) protein in articular cartilage following ischemic osteonecrosis (ON) induction in a mouse model of juvenile ischemic ON. (*A*) HMGB1 protein was immunohistochemically analyzed with anti‐HMGB1 antibody. HMGB1 immuno‐staining intensity was much higher in the articular cartilage after ischemic induction at day 7 in the ON‐right group compared with the sham‐right group. The HMGB‐1 immuno‐staining was not detectable in the contralateral ON‐left and sham‐left groups (ON: *n* = 4; sham: *n* = 4). R = Right side; L = left side. Bars: 50 μm. (*B*) HMGB1 protein level was analyzed by Western blot. After 24 hours of ON or sham surgery, the articular cartilage of the distal femur was harvested, and protein was extracted. HMGB1 protein was highly expressed in the ON‐right group compared with the sham‐right group (ON: *n* = 4; sham: *n* = 4). Note that HMGB1 protein was faintly expressed in the contralateral sides (ie, sham‐left, ON‐left). R = Right side; L = left side.

We reported that articular chondrocytes increased IL‐6 gene expression and protein production using an experimental mouse model of juvenile ischemic ON.^(^ ^39^ ^)^ Using the same mouse model, we found significantly increased HMGB1 protein immunostaining in the nucleus, cytoplasm, and some extracellular space of the articular chondrocytes in the ON group compared with the sham group (ie, ON‐right versus sham‐right) at postischemia day 7 (Fig. 4*A*). In the normal and sham‐operated sides, HMGB1 immunostaining was mostly negative (Fig. 4*A*). We harvested the articular cartilage and determined HMGB1 protein level by Western blot. The increase of HMGB1 protein in the ON group was confirmed compared with the sham group (ie, ON‐right versus sham‐right) at postischemia day 1 (Fig. 4*B*). Further, HMGB1 gene expression in the ON‐right side was increased by sixfold compared with the sham‐right side at postischemia day 1 (Supplemental Fig. S1).

### Hypoxic and oxidative stresses increased HMGB1 and IL‐6 in human chondrocytes

**Fig 5:**
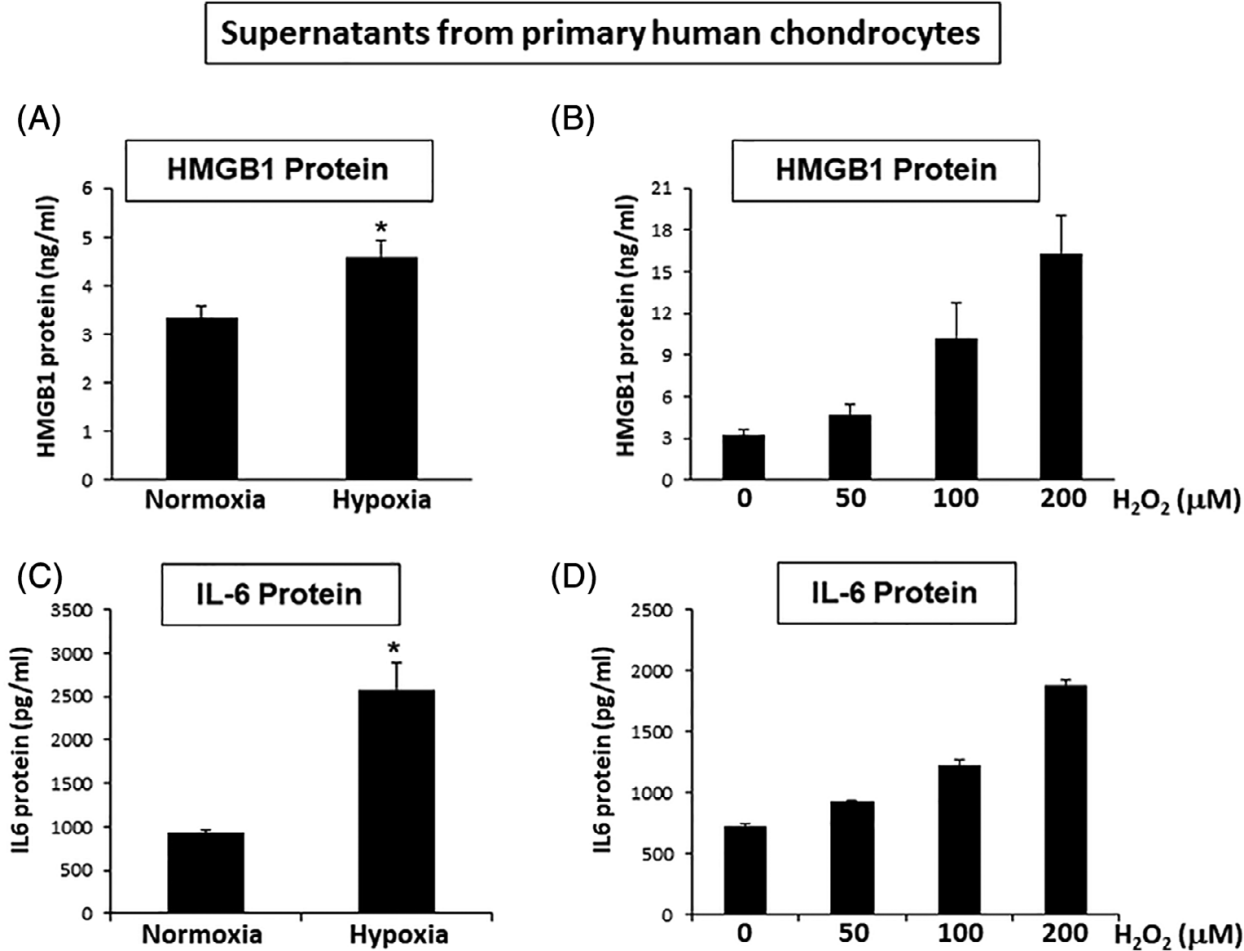
Chondrocytes exposed to hypoxic and oxidative stresses produced high‐mobility group box 1 (HMGB1) and IL‐6 proteins in culture supernatant. (*A*) Human primary chondrocytes were cultured in normoxia or hypoxia for 24 hours, and culture supernatant was collected from each condition. The concentration of HMGB1 protein was significantly higher in the supernatant of hypoxic than that of normoxic condition. *n* = 3 per each group, *p < 0.05. (*B*) Hydrogen peroxide (H_2_O_2_; 0 to 200μM) was added to the culture of primary chondrocytes for 24 hours. The concentration of HMGB1 protein in the culture supernatants was increased in a dose‐dependent manner. (*C*) Human primary chondrocytes were cultured in normoxia or hypoxia for 24 hours, and culture supernatants were collected from each condition. The concentration of IL‐6 protein was significantly higher in the supernatant of hypoxic than that of normoxic condition. *n* = 3 per each group, *p < 0.05. (*D*) H_2_O_2_ (0 to 200μM) was added to the culture of primary chondrocytes for 24 hours. The concentration of IL‐6 protein in the culture supernatants was increased in a dose‐dependent manner.

Hypoxic stress on chondrocytes can induce passive release of HMGB1 protein in extracellular space.^(^ ^24^ ^)^ We next isolated primary human chondrocytes from articular cartilage and confirmed that HMGB1 protein levels were significantly higher in the culture supernatant under hypoxic than that under normoxic condition (Fig. 5*A*). Under this condition, IL‐6 protein levels were also increased in the culture supernatant (Fig. 5*C*). Hypoxia also induces oxidative stress via reactive oxygen species (ROS) such as H_2_O_2_.^(^ ^45^ ^)^ H_2_O_2_ stimulates immune cells to release HMGB1.^(^ ^48^ ^)^ Here, we found that the oxidative stress on chondrocytes by H_2_O_2_ increased HMGB1 protein levels in the culture supernatants in a dose‐dependent manner (Fig. 5*B*), and that IL‐6 protein levels were similarly increased in the culture supernatants (Fig. 5*D*).

### Possible relationship between HMGB1 and IL‐6 in human chondrocytes in vitro

**Fig 6:**
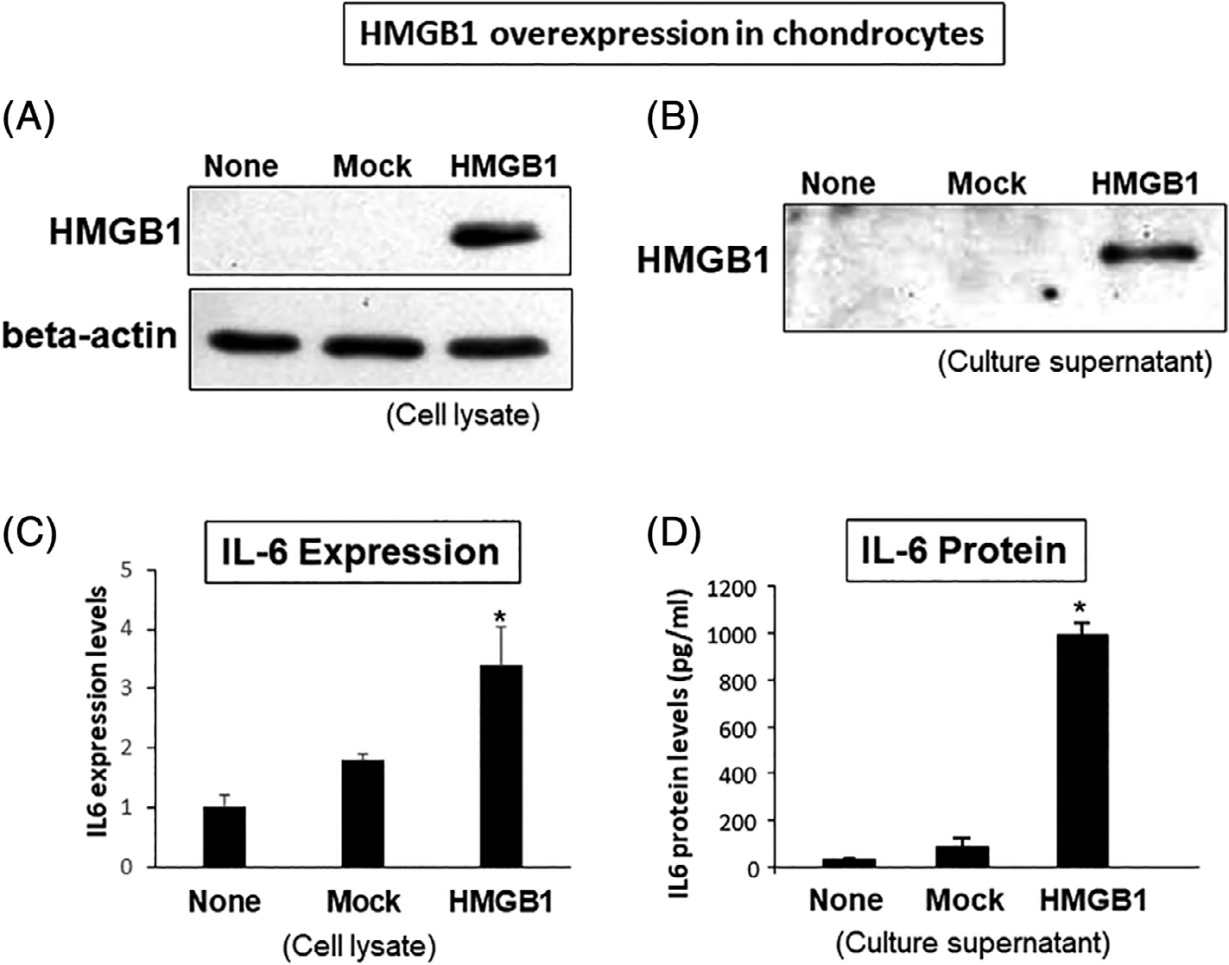
Overexpression of high‐mobility group box 1 (HMGB1) protein in the culture supernatant using chondrocytes. (*A*) ATDC5 cells were transfected with HMGB1 or mock plasmid and cultured for 48 hours. Cells were harvested and protein was extracted. HMGB1 protein was detectable in the HMGB1‐transfected cells; it was undetectable in the mock‐transfected cells. Representative image is shown (*n* = 3 per each group). (*B*) HMGB1 protein was detectable in the culture supernatants from HMGB1‐transfected ATDC5 cells; it was undetectable in the mock‐transfected culture supernatants, as derived from the culture represented by Fig. 6*A*. Representative image is shown (*n* = 3 per each group). (*C*) Human chondrocytes were transfected with HMGB1 or mock plasmid and cultured for 48 hours. Expression levels of IL‐6 in the HMGB1‐transfected cells were significantly higher than that in the mock‐transfected cells, as derived from the culture represented in Fig. 6*A*. *p < 0.05 (mock: *n* = 4; HMGB1: *n* = 4). (*D*) Human chondrocytes were transfected with HMGB1 or mock plasmid and cultured for 48 hours, and culture supernatants were collected from each condition. The concentration of IL‐6 protein was significantly higher in the HMGB1‐transfected supernatants than that in the mock‐transfected supernatants. *p < 0.05 (mock: *n* = 4; HMGB1: *n* = 4).

To investigate active secretion of HMGB1 from chondrocytes, we first overexpressed endogenous HMGB1 protein in human cell‐line 293 cells (Supplemental Fig. S2). Next, we applied this condition to mouse chondrocyte cell‐line ATDC5 cells and human primary chondrocytes. When HMGB1 protein was overexpressed in the ATDC5 cell lysate (Fig. 6*A*), HMGB1 was detectable in its culture supernatant (Fig. 6*B*). Using human primary chondrocytes, we found that the IL‐6 RNA level was significantly higher in the HMGB1‐transfected cells compared with that in the mock‐transfected cells (Fig. 6*C*). The IL‐6 protein level was also significantly higher in the culture supernatant from the HMGB1‐transfected cells compared with that from the mock‐transfected cells (Fig. 6*D*).

**Fig 7:**
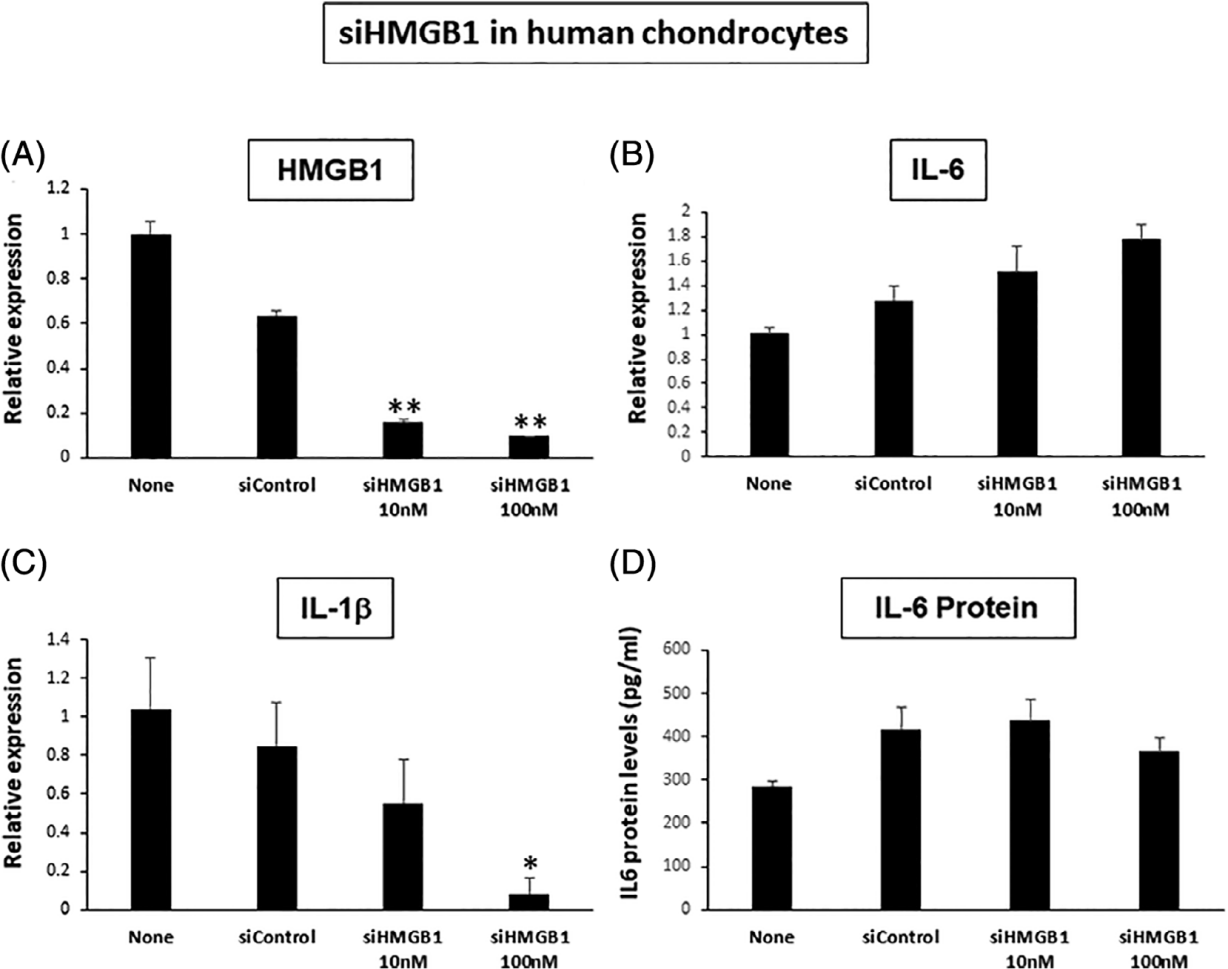
Inhibition of high‐mobility group box 1 (HMGB1) by short interfering RNA (siRNA) in human chondrocytes. (*A*) HMGB1 expression was significantly reduced by 90% in the siHMGB1‐transfected cells compared with the siControl‐transfected cells. The value from the nontreatment cells (ie, None) was set as 1.0. **p < 0.01. (*B*) IL‐6 expression was unchanged between the siHMGB1‐transfected and siControl‐transfected cells. (*C*) IL‐1β expression was significantly reduced by 91% in the siHMGB1‐trenasfected cells compared with the siControl‐transfected cells. *p < 0.05. (*D*) The concentration of IL‐6 protein in the supernatants was unchanged between the siHMGB1‐transfected and siControl‐transfected cells (*n* = 3 per each group in the Fig. 7).

We further conducted a loss‐of‐function experiment by silencing *HMGB1* using a siRNA method in human chondrocytes. The HMGB1 expression levels were significantly reduced by about 85% in the siHMGB1‐transfected cells compared with the siControl‐transfected cells (Fig. 7*A*). Under this silencing efficiency, IL‐1β gene expression was significantly reduced by over 90%, suggesting the transcriptional regulation of IL‐1β gene expression by *HMGB1* **(**Fig. 7 *C* **)**. However, IL‐6 gene expression in the cultured cells and protein levels in the culture supernatants were both unchanged (Fig. 7*B*,*D*).

### Possible relationship among IL‐6, IL‐1β, and TNF‐α in the LCPD synovial fluid

**Fig 8:**
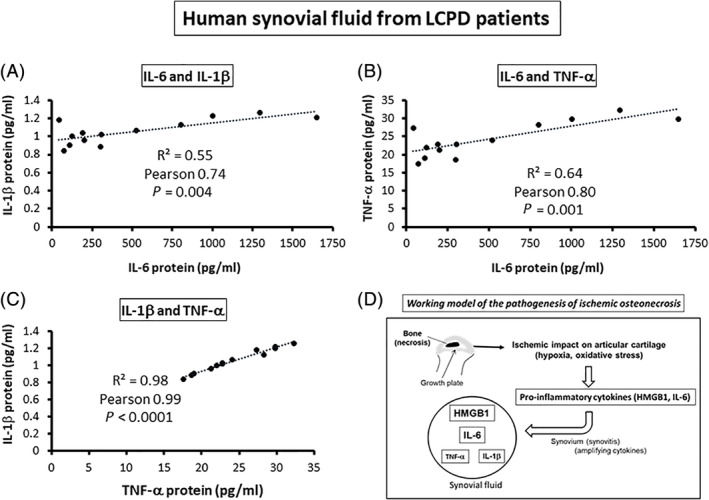
Positive correlations among IL‐6, IL‐1β, and TNF‐α protein levels in the synovial fluid of patients with Legg‐Calvé‐Perthes disease (LCPD). (*A*–*C*) Protein levels of IL‐6, IL‐1β, and TNF‐α (pg/mL) in synovial fluid of patients with LCPD (*n* = 13) were measured by a multicytokine assay kit. Each scatter plot shows one human sample. Significant correlations were observed between IL‐6 and IL‐1β (Pearson correlation coefficient 0.74, *p* = 0.004, *R* ^2^ = 0.55), between IL‐6 and TNF‐α (Pearson correlation coefficient 0.80, *p* = 0.001, *R* ^2^ = 0.64), and between IL‐1β and TNF‐α (Pearson correlation coefficient 0.99, *p* < 0.0001, *R* ^2^ = 0.98). (*D*) A possible model of disease mechanism following ischemic osteonecrosis. Proinflammatory cytokines high‐mobility group box 1 (HMGB1) and IL‐6 were produced by articular chondrocytes following ischemic induction and were amplified via synovium, resulting in the increase of cytokines in the synovial fluid including IL‐1β and TNF‐α.

Protein concentrations of IL‐6, IL1‐β, and TNF‐α in the synovial fluid of patients with LCPD (*n* = 13) were measured using a multicytokine assay kit. Here, we found significant correlations in the levels of proinflammatory cytokines between IL‐6 and IL‐1β (Pearson correlation coefficient 0.74, *p* = 0.004, *R* ^2^ = 0.55), between IL‐6 and TNF‐α (Pearson correlation coefficient 0.80, *p* = 0.001, *R* ^2^ = 0.64), and between IL‐1β and TNF‐α (Pearson correlation coefficient 0.99, *p* < 0.0001, *R* ^2^ = 0.98; Fig. 8*A*–*C*).

## Discussion

### Overall findings

Although the clinical manifestation of LCPD was first described over 100 years ago, there is no effective medical therapy or biological agents currently available for patients with LCPD. In this study, we first report that the HMGB1 protein, a proinflammatory cytokine, was significantly elevated in the synovial fluid of patients with LCPD. This finding is clinically relevant as HMGB1‐targeting therapeutic strategies have been developed for other diseases.^(^ ^13^, ^49^ ^)^

The current study showed a significant increase in HMGB1 protein and gene expression levels in the articular chondrocytes following ischemic ON in the mouse model. HMGB1 protein was released in the supernatants of human chondrocytes when exposed to hypoxic (ie, 1% oxygen) and oxidative (ie, H_2_O_2_) stresses in vitro, concomitant with IL‐6 elevation. Similarly, HMGB1 protein was secreted in the supernatants of chondrocytes with increased IL‐6 production. A loss‐of‐function experiment in vitro using siHMGB1 showed a significant reduction in IL‐1β gene expression, but not for IL‐6. In addition to HMGB1, both IL‐1β and TNF‐α protein levels were significantly correlated with IL‐6 protein levels in the synovial fluid of patients with LCPD. Based on these findings, we can postulate a possible working model **(**Fig. 8 *D* **)** that articular chondrocytes following ischemic ON respond to hypoxic and oxidative stresses by producing HMGB1 in addition to IL‐6, and that other proinflammatory cytokines, IL‐1β and TNF‐α, are also involved in the pathogenesis of LCPD with significant correlation to IL‐6 in the synovial fluid.

### Clinical significance

In last 20 years, more and more biological agents have been clinically incorporated in the treatment of bone diseases, including osteoporosis, RA, and juvenile idiopathic arthritis. For RA, about 10 biological agents are currently available by targeting specific molecules involved in the disease mechanisms of chronic synovitis at different molecular target (ie, ligand, receptor), inflammatory cytokine cascade (ie, IL1‐β, TNF‐α, and IL‐6), and cell type (ie, B cell, T cell). Currently, the effectiveness of a biological agent differs from patient to patient,^(^ ^50^ ^)^ probably because of the difference in multifaceted inflammatory mechanisms and disease progression. Thus, it would be beneficial to develop multiple therapeutic agents for one disease and establish comprehensive protocols for patients based on the complexities of the disease.

As mentioned above, multiple therapeutic strategies are desired by the targeting of the different molecules involved in the disease mechanisms of LCPD. The current study highlight HMGB1 as another candidate drug target in addition to IL‐6. HMGB1 is known to be elevated in many diseases, including autoimmune disease.^(^ ^13^ ^)^ RA is the first confirmed autoimmune disease linking HMGB1 to immunomediated conditions.^(^ ^51^, ^52^ ^)^ Therefore, the importance of HMGB1 as a biomarker and a therapeutic target has been described,^(^ ^13^, ^49^, ^53^, ^54^, ^55^ ^)^ and several putative approaches of HMGB1 inhibition (ie, antagonist, neutralizing antibody, and small molecule inhibitor) have been investigated.^(^ ^13^ ^)^ In patients, the increase of HMGB1 in serum and synovial fluid has been reported as a therapeutic target of juvenile idiopathic arthritis,^(^ ^56^ ^)^ RA,^(^ ^57^, ^58^, ^59^ ^)^ and OA.^(^ ^27^, ^28^ ^)^ In an experimental study, the injection of HMGB1 protein into a normal joint induced arthritis in 80% of mice.^(^ ^29^ ^)^ Furthermore, HMGB1 inhibition by the administration of anti‐HMGB1 antibodies and HMGB1 antagonist had cartilage‐protective effects, and prevented the progression of arthritis in other studies.^(^ ^60^, ^61^, ^62^ ^)^ Interestingly, no arthritis was induced when HMGB1 protein was injected into IL‐1‐receptor–deficient mice,^(^ ^29^ ^)^ suggesting a functional requirement of IL‐1 for the HMGB1‐induced arthritis.

In the current study, we found significant positive correlations among TNF‐α, IL‐1β, and IL‐6 proteins in the synovial fluid of patients with LCPD (Fig. 8). In the previous study, however, both TNF‐α and IL‐1β were not elevated compared with controls,^(^ ^32^ ^)^ probably because both TNF‐α and IL‐1β are early cytokines that may return quickly to baseline levels.^(^ ^63^ ^)^ In RA, both TNF‐α and IL‐1β are elevated; thus they are therapeutic targets. In addition, clinical manifestations are very different between LCPD and RA (ie, local versus systemic, male > female versus male < female, etc, respectively), indicating a distinct pathophysiology of LCPD from RA.

### Extracellular HMGB1 protein from chondrocytes

Extracellular roles of HMGB1 were first described in 1999.^(^ ^21^ ^)^ HMGB1 acts as a proinflammatory cytokine^(^ ^14^, ^15^, ^16^ ^)^ with a positive feedback loop by synergistically increasing proinflammatory cytokines IL‐6, IL‐1β, and TNF‐α in immune cells.^(^ ^19^, ^20^, ^21^, ^22^, ^23^ ^)^ Neutralizing antibody for HMGB1 was found to reduce serum levels of IL‐6, IL‐1β, and TNF‐α in rat.^(^ ^64^ ^)^ In fact, IL‐1β stimulates chondrocytes to induce the translocation of HMGB1 from the nucleus to cytoplasm and to increase extracellular HMGB1.^(^ ^25^ ^)^ In turn, HMGB1 also stimulates chondrocytes to produce IL‐1β and TNF‐α in culture supernatants.^(^ ^25^ ^)^

In the current study, articular chondrocytes are a putative source to produce HMGB1. This is because HMGB1 protein was translocated into cytoplasm and some to extracellular space of articular chondrocytes following ischemic induction in the mouse model (Fig. 4). HMGB1 protein was elevated in the culture supernatants when human chondrocytes were exposed to hypoxic and oxidative stresses (Fig. 5) and were transfected with the plasmid for HMGB1 overexpression (Fig. 6). These results are consistent with other studies that showed abundant HMGB1 protein in the cytoplasm from damaged articular chondrocytes of patients with OA,^(^ ^25^, ^26^ ^)^ and in the culture supernatants from chondrogenic cell‐line ATDC5 cells exposed to a hypoxic stress.^(^ ^24^ ^)^ In addition, articular chondrocytes in OA cartilage express the receptor for advanced glycation end products (RAGE), a specific receptor for HMGB1,^(^ ^26^, ^65^ ^)^ postulating a common feature of LCPD and OA with autocrine loop to amplify HMGB1 via chondrocytes.

We have shown that hypoxia‐inducible factor‐1α (HIF‐1α) protein was significantly increased in the articular cartilage of the femoral head following the induction ischemic ON in a piglet model of LCPD,^(^ ^66^ ^)^ and that IL‐6 can be activated in articular chondrocytes in a HIF‐1α dependent manner.^(^ ^40^ ^)^ Interestingly, HMGB1 enhances HIF‐1α mRNA levels and activities in synovial fibroblasts from patients with RA,^(^ ^67^ ^)^ and the association of HMGB1 and HIF‐1α has been found in other diseases including cancer.^(^ ^68^ ^)^ The regulation of HMGB1 by HIF‐1α is unknown. Further study is needed to investigate the role of HMGB1 in the regulation of HIF‐1α and vice versa in articular chondrocytes following ischemic ON.

### Limitations of this study

There are some limitations in this study because of the nature of HMGB1. HMGB1 overexpression increased IL‐6 expression; however, the loss‐of‐function experiment for HMGB1 did not reduce IL‐6 expression, but repressed IL‐1β expression. Although this result is consistent with the evidence that IL‐1β is an extracellular binding partner for HMGB1,^(^ ^20^, ^42^, ^69^, ^70^, ^71^ ^)^ IL‐6 production can be compensated by an unknown mechanism that was not elucidated in this study. A follow‐up study is warranted as toll‐like receptor 2, one of putative receptors for HMGB1, was significantly elevated when HMGB1 was diminished (data not shown). In addition, the direct effect of HMGB1 on synovitis should be investigated using animal models of ischemic ON where anti‐HMGB1 antibodies are administered.

### Conclusion

The importance of extracellular HMGB1 protein as a proinflammatory cytokine has been reported in many diseases. In this study, we first revealed that HMGB1 protein was elevated and significantly correlated with IL‐6 protein in the synovial fluid of patients with LCPD. Articular chondrocytes respond to the induction of ischemic ON by producing HMGB1 in addition to IL‐6. Other proinflammatory cytokines, IL‐1β and TNF‐α, are also involved in the pathogenesis of LCPD with significant correlation to IL‐6. This study is clinically relevant because therapeutic strategies for these cytokines may be applicable to the development of an effective medical therapy for LCPD.

## Disclosure

All authors state that they have no conflicts of interest.

### PEER REVIEW

The peer review history for this article is available at https://publons.com/publon/10.1002/jbm4.10429.

## Supporting information


**Supplemental Figure S1** Increase of HMGB1 gene expression in the articular cartilage using an experimental mouse model of juvenile ischemic osteonecrosis. After 24 hours of osteonecrosis or sham surgery, the articular cartilage of distal femur was harvested and mRNA was isolated. Expression levels of HMGB1 in the ON‐right group were significantly higher than that in the sham‐right group. The value of contralateral sham‐left group was set as 1.0. *p < 0.05 (ON; *n* = 4, sham; *n* = 4), R; right side, L; left side

**Supplemental Figure S2** Overexpression of HMGB1 in 293 cells. 293 cells were transfected with HMGB1 or mock plasmid and cultured for 48 hours. Cells were harvested and protein was extracted. HMGB1 protein was detectable in the HMGB1‐transfected cells while it was undetectable in the mock‐transfected cells. Representative image is shown (*n* = 3 per each group).
