# Supplementary figures and images for: Elevation of Proinflammatory Cytokine HMGB1 in the Synovial Fluid of Patients With Legg‐Calvé‐Perthes Disease and Correlation With IL‐6
Source: JBMR Plus. 2020 Dec 3;5(2):e10429. doi: 10.1002/jbm4.10429 (PMC7872337; doi:10.1002/jbm4.10429)

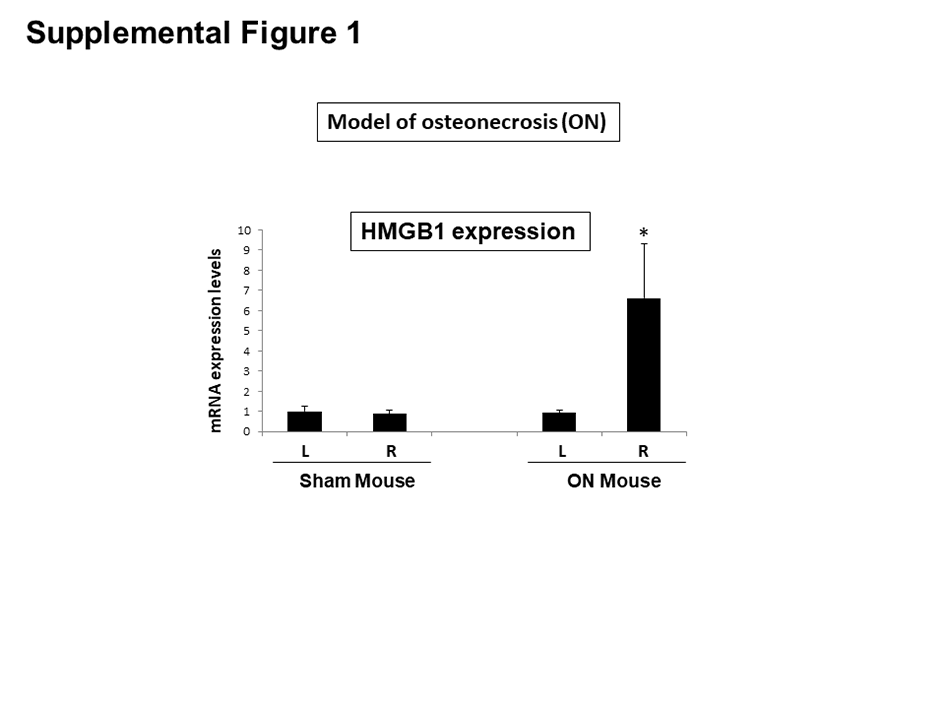

Supplement: Supplementary file 1 — Supplemental Figure S1 Increase of HMGB1 gene expression in the articular cartilage using an experimental mouse model of juvenile ischemic osteonecrosis. After 24 hours of osteonecrosis or sham surgery, the articular cartilage of distal femur was harvested and mRNA was isolated. Expression levels of HMGB1 in the ON‐right group were significantly higher than that in the sham‐right group. The value of contralateral sham‐left group was set as 1.0. *p < 0.05 (ON; n = 4, sham; n = 4), R; right side, L; left side [file JBM4-5-e10429-s001.tif]

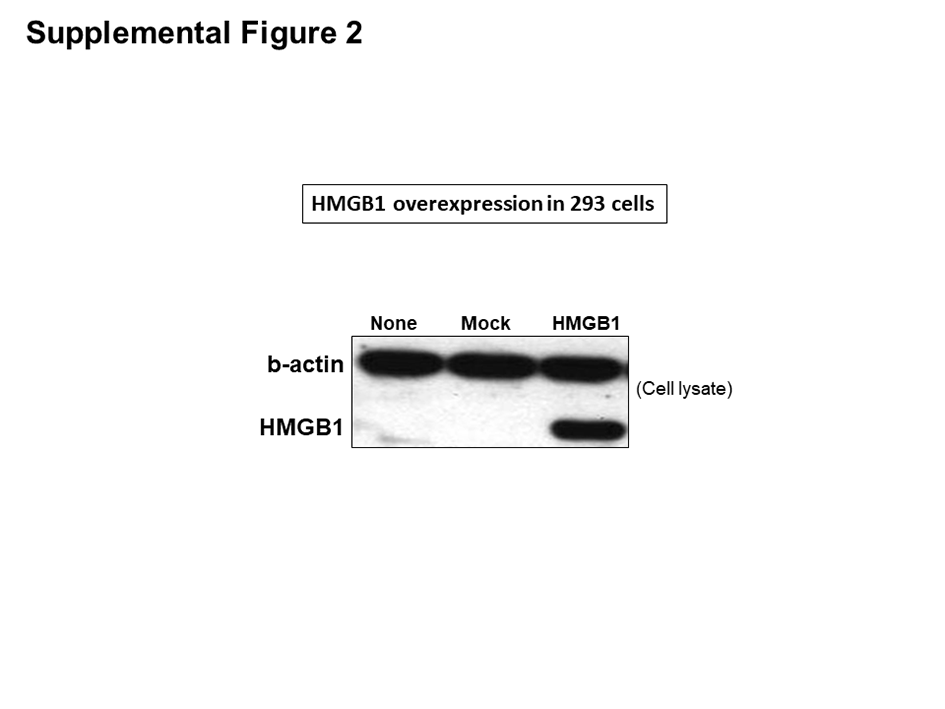

Supplement: Supplementary file 2 — Supplemental Figure S2 Overexpression of HMGB1 in 293 cells. 293 cells were transfected with HMGB1 or mock plasmid and cultured for 48 hours. Cells were harvested and protein was extracted. HMGB1 protein was detectable in the HMGB1‐transfected cells while it was undetectable in the mock‐transfected cells. Representative image is shown (n = 3 per each group). [file JBM4-5-e10429-s002.tif]
